# Case of Deformed Leg, from Unsuccessfully Treated Fracture, Cured by an Operation Performed by John Rhea Barton, M. D.

**Published:** 1842-02

**Authors:** W. S. W. Ruschenberger

**Affiliations:** U. S. Navy


*Case of Deformed Leg, from unsuccessfully treated Fracture, cured by an operation performed by* John Rhea Barton, M. D.—Reported by W. S. W. Ruschenberger, M. D., U. S. Navy.—About half past seven o'clock, P. M., on the 18th of December, 1838, while in charge of the deck of the U. S. Ship Ohio, then at sea, Lieutenant —— fell from the horse block, about four feet high. The weather being cold and boisterous, he was heavily clothed in a pea-jacket, &c. His feet became engaged in a coil of rigging on the deck, while the body was carried forward, and the right tibia was fractured transversely at about its lower third, and the fibula at about two and a half inches above the ankle.

A gale of wind, then commencing, lasted several days.

He was placed in his apartment on the orlop deck, where the circulation of air was very much interrupted, and probably much vitiated by the number of persons breathing it.

The limb was set by the surgeon. He suffered very much from the motion of the ship, and in the night was attacked with severe spasms in the limb; and he distinctly felt the fragments slip upon each other.

On the 5th of January, 1839, the ship arrived at Mahon, and on the following day, the nineteenth after the accident, the patient was moved on shore. During the transportation from the ship, he suffered great pain from the moving of the ends of the broken bones on each other.

He remained in bed eight weeks, and when he got up, the limb was still flexible at the point of fracture. Getting out of bed was at first very painful, and usually occupied fifteen minutes.

When he got up he was urged to exercise the limb, and three or four weeks afterwards, to bear his weight, in a degree, upon it.

In consequence of the accident, and its unsuccessful treatment, the upper fragment of the tibia rides the lower one, overlapping it about half an inch, forming an obtuse angle which presents inwards. The limb is shortened a half inch; there is a concavity inwards, on the outside of the leg, as might be the case if the fibula were pressed inwards against the tibia at its lower third; the flexor muscles are thrown out of their normal line of action, and the external condyle of the femur seems to be, in a measure, alone in the essential constitution of the knee-joint, the internal ligaments being elongated, and the knee thrown inwards.

The patient suffers no pain; and the only inconvenience complained of is, that his footing is not certain when at sea, and that he suffers under pain and weakness in the knee, on taking unusual exercise on shore.

For the purpose of removing this inconvenience, and correcting the deformity, the patient came to Philadelphia; and after having been carefully examined at different times, and at considerable intervals, by Doctors Thomas Harris, W. E. Horner, J. Randolph, W. S. W. Ruschenberger, J. Rhea Barton, and Paul B. Goddard, anxiously submitted to an operation.

Lieutenant ——— is a native of South Carolina; he is thirty-four years of age, about five feet eight inches high, of nervous sanguine temperament, light eyes, ruddy complexion, and, with the exception of an attack of fever on the coast of Africa, in 1824, has enjoyed uninterrupted health. He does not use tobacco in any form.

For a month he has regulated his diet with a view to the operation, eating moderately of meat once a day.

Having procured an airy, comfortable apartment, and made the necessary preparations, he submitted to the following operation, performed by J. Rhea Barton, assisted by Doctors Norris, E. Peace, Paul B. Goddard, W. P. C. Barton, and Ruschenberger.

*October* 18th, 1841. Weather clear and cool. Ten minutes before commencing the operation the patient swallowed thirty-five drops of laudanum. He was placed upon the table at twelve o'clock.

Two incisions, three inches in length, were made over and parallel with the internal and external margins of the tibia, three inches apart at their upper extremities, and two and a half at their termination. These two incisions were connected by a transverse cut, made a little below the nearly square projecting end of the upper fragment of the tibia; the three incisions describing the letter H. The flaps thus formed, consisting of the skin and subjacent cellular tissue only, were raised up, exposing the fragments of the tibia at their point of union. The adjacent muscles were separated from the bone by the handle of the scalpel; the periosteum, very near the lower termination of the upper fragment, was divided by the scalpel ; a small saw, somewhat in the form of a carving-knife, about ten inches long in the blade, suddenly tapered into a point of two and a half inches long, and rounded at the extremity, was next employed, and a slice of bone, less than a line in thickness, removed from the extremity of the upper fragment. The saw was carefully worked in the same line of direction, and in the same plane, frequently removing it to clear its teeth by a sponge, until the lower fragment was divided nearly through. What remained was forcibly fractured—a short, stout spiculum, adhering to the posterior portion of the lower fragment, and which was afterwards removed.

Upon examination, it was now found, as was anticipated, that transverse bridges of bone connected the tibia and fibula together above and below the seat of fracture—having been formed there after the injury, for the wise purpose of supporting the weakened limb—and prevented the upper and projecting portion from being brought in a normal line with the lower fragment. These bony bridges were removed by the aid of a chisel and strong nippers: and, by the same means, the ends of the two fragments of the tibia were adjusted and finally brought into perfect coaptation. The operation occu-occupied nearly an hour. No vessel required ligature ; and the loss of blood did not exceed eight ounces.

The edges of the wound were brought together, and retained by adhesive straps. Lint, spread with simple cerate, was placed over them. The limb, from the toes to the knee, was then covered by successive turns of a roller. The patient was now carefully removed to bed. A soft, square pad, two inches thick, was placed over the external maleolus, and a similar one close to the knee-joint ; upon these was laid a splint two and a half inches wide, to which the two fragments of the tibia were confined by a few turns of a roller, applied at the proper points. A soft pillow, covered with oiled silk, was made to half encircle the limb, by the aid of splints in a splint cloth, and the whole secured by tapes. The limb was protected from the pressure of the bed-clothes in the usual way.

Directed to take in the evening a cup of weak black tea, and a piece of dry toast.

9 o'clock, P. M. Patient comparatively comfortable.

19*th.*—9 o'clock, A. M. Skin natural; very slight suffusion of countenance; bowels not moved; pulse 80, and soft. Had very little sleep since the operation; experienced some few "twinges" in the limb. Became comparatively easy an hour after the operation. Complains of not being able to relax the muscles of the limb. Pain in the back last night and this morning.

A compress placed over the upper fragment, near its inferior extremity, to prevent its rising; it is secured in its place by tying one of the tapes of the splints across it.

Diet.—Barley water, chicken broth.

6 o'clock, P. M. Comfortable. Felt oppressed after taking broth; relieved after passing urine and perspiring freely. Urine brandy colored.

20*th.*—9 o'clock, A. M. Pulse 76; skin comfortable; looks cheerful. At 4 o'clock, A. M. had severe continued pain in the abdomen, probably from flatus. Patient applied hot dry cloths with advantage. Tongue slightly white.

Diet.—Small piece of roast beef; barley water.

9 o'clock, P. M. Has not been able to have an evacuation from the bowels from want of convenience, as he cannot get a pan beneath him, and is insurmountably repugnant to the use of a sheet. Complains that he has passed a miserable day; belly very tender and sore, but it is soft and not distended; pulse 76. Moved to a mattrass with a moveable piece on the left side, corresponding to the pelvis. The moving caused great pain and considerable exhaustion.

21*st.*—9 o'clock, A. M. After taking some warm broth the bowels were moved; and he has had another evacuatfon this morning. Slept several hours. Soreness and tenderness of the abdomen have disappeared; pulse 76; comfortable; says he is not aware, from any uneasy feeling, that his leg is broken.

External soiled parts of the dressing removed by scissors, and a Scultetus bandage placed over the whole; the oiled silk changed for a clean piece.

Diet continued.

6 o'clock, P. M. Had spasm in the leg in the afternoon.

22*d.*—Half past 9 o'clock, A. M. Pulse 76, and after taking coffee, (which acts kindly on his bowels,) 79. Had very little sleep last night; some spasms; bowels open.

Removed the dressings. Wound healthy.

A number of maggots, nearly a half inch long, about the wound. Washed with Castile soap and warm water. Dressing of lint and simple cerate. Pads at the ankle and knee removed, and the splint, lightly padded, brought in contact with the limb. From a slight thickening of the lower flap, the lower fragment of the tibia *seems* to be in advance of the upper one.

Continue diet.

6 o'clock, P. M. No sleep; some spasm; great pain in the foot from pressure of the bandages, which was relieved by removing and re-applying them. Wound looks well.

Liquor anodyn. Hoffman. H. S.

23*d*.—Half past 9 o'clock, A. M. Slept about five hours last night; bowels open; pulse 76. Continue treatment.

6 o'clock P. M. Has had a comfortable day.

24*th*.—Half past 9 o'clock, A. M. Slept four hours; comfortable; pulse 76; bowels open; urine high colored, but now transparent. Continue treatment.

27*th*.—Nothing remarkable since last report. Slept very little last night. Wash the wound with tinct. myrrh.

Continue diet; half a tumbler of Philadelphia brown stout.

28*th*.—Slept six hours. Bran bags applied. Continue treatment.

*November* 2*d*.—In consequence of the upper fragment having slightly fallen out its proper line, a straight, narrow splint was applied on the outside of the leg.

5*th*.—Paste-board splint applied to the inside of the leg; bone begins to stiffen.

6*th*.—Splint applied on the inside of the leg, which reposes on the pillow without being secured by the splints and splint-cloth. Bowels are open every morning; pulse 80; sleeps four or five hours in the twenty-four; is very cheerful.

7*th*.—Wound nearly healed; suppuration has almost ceased.

*December* 31*st*.—At present the patient is walking about his apartment, aided by a cane. He wears the same boots that he wore previous to the operation, and does not think the limb is appreciably shortened. At the end of a fortnight he will be able to travel; but at this time it would be premature to state what the effect of the operation has been, although the limb is straight, and not appreciably shorter than it was prior to the operation.

To the young practitioner of surgery, this case is particularly interesting, and should lead all to study carefully the treatment of fractured bones, the result of which, if successful, adds very little to the reputation of the surgeon, but, if otherwise, is calculated to injure his professional character.

The following short notes of this case, were made by the patient himself, and are given in his own words:

*October 18th*, the day of the operation. The *cutting* less painful, and sawing more so than I had anticipated, especially on some portions of the bone, which were more sensitive than other portions. The use of the hammer and chisel to separate extraneous masses of bone, excessively shocking to the nervous system. It occupied rather more than one hour ere I was placed in bed and the limb adjusted, and three-quarters of an hour under the actual operation.

On being put to bed, and the wound dressed, I had little or no pain, and in every sense became comfortable. What pain there was, occurred at intervals of half to three-fourths of an hour, lasting only a few minutes, of a burning sensation rather than what I should call acute pain, and which seemed to linger about the exterior. Pulse rather full till nine P. M., when a healthy re-action took place. No symptoms of fever. The night was sleepless; little pain, but continued thirst.

*Second day*. The pains diminishing, and the intervals between them increased. Good spirits: no fever. An acute soreness in the abdomen, with excessive pain in the back—doubtless the effect of position. Had an hour's sleep. During the night obtained three hours sleep; but suffered greatly with pain in the abdomen and from thirst.

*Third day*. Little or no pain in the limb. Soreness in back and abdomen continuing; spirits good; no fever; little appetite. During the night had three hours sleep; much thirst; and always more nervous during the night than day.

*Fourth day*. Pain in abdomen and back very much abated, and the whole system more agreeable than heretofore. Appetite better. No fever. To-day the wound was exposed, and pronounced to have a healthy appearance. Though the day was agreeable, yet the night was the most irksome and painful of any previous to it, caused by a spasmodic attack in the limb at eleven P. M., and repeated several times during the night, occasioning an unusual flow of blood, creating pricking sensations about the wound and in the calf, from which I was relieved when the wound was dressed in the morning. It was remarked to-day that the muscles seemed to have accommodated themselves easily to their present and more natural position, from which they had been diverted just *two years and ten months;* and the bones retained in the desired position by very slight means.

*Fifth day.* After the dressing, the limb became easy: the *back* and *abdomen* are entirely relieved from pain at last. No fever, and spirits good. Several spasms occurred.

Had five hours sleep and no spasm.

*Sixth day.* No actual pain, but the wound is becoming extremely sensitive to touch or motion. The foot benumbed and heavy, and subject to frequent change from heat to cold, and vice versa. Digestion good. Four hours sleep, and no spasm.

*Seventh day.* The sensitiveness of the wound increasing, but still appears healthy; (what the doctors call beautiful.) The foot becoming more and more weighty, but has not swollen in the least.

Had four hours sleep at night; spasms abated.

*Eighth day.* Differs in no degree from the preceding one.

*Ninth day.* The wound has now become unbearably sensitive to the slightest touch or motion—ridiculously so. The spring of the floor, occasioned by a person walking across the room, and even the jar communicated to the building by carriages driving along the paved streets, affecting (if not actually afflicting) the wounded parts. In the first instance the pain was confined to the exterior wound, but now the interior has more than its share. Especially where the extraneous bones or bridges were removed is the seat of inconvenience.

*Tenth day.* No change in the last twenty-four hours.

*Eleventh day.* The same as the preceding twenty-four hours.

*Twelfth day.* No alteration in the sensitiveness of the wounded parts. At 5 P. M. was seized with a severe pain in the foot, as if all the bones in it had been crushed by some weighty mass. At six it became excruciating; caused it to be rubbed with oil of camphor, which allayed the pain almost immediately.

*Thirteenth day.* Extreme sensitiveness of the wound somewhat abated; otherwise no pain.

*Fourteenth day.* The transitions from heat to cold, &c., benumbed feeling, and sense of weight in the foot, mentioned on the sixth day, have continued up to this time, but at length are abating, together with the sensitiveness in the region of the wound.

From the second to the end of the third week, no new symptoms. No swelling. Good health. All inconveniences gradually diminishing. The union of the bones considered to have occurred unusually rapid during this week. On the twenty-first day it was considered tolerably firm. The only pain and inconvenience to which I am now subjected, are from the application of *splints*, *compresses*, and such means as are usual for retaining the bones in their proper place.

To the end of the fourth week little or no pain, or other inconvenience consequent upon the operation. The union of the bones has become so firm as to admit of the limb being rolled about the pillow, and the wound healed, except in one point, where it is frequently probed, a fragment of bone being about to detach itself.

To the end of the fifth week, the union hardening perceptibly each day. Left the bed several times this week, for a few minutes, to receive a cold shower upon the limb. On the fortieth day, arose from the bed on crutches, and in four days thereafter the splints and bandages were dispensed with.

Remarks—During my confinement to bed, the pulse ranged from 70 to 87; and sometimes, from exciting causes, as high, temporarily, as 93. But my pulse is usually rapid, and easily excited. My appetite did not vary from its customrry moderate standard.

Entertaining no anxiety for the result, my mind was calm; and I was even satisfied with my confinement and almost torpid state of body.

It was remarked that the greatest degree of uneasiness, pains, spasms, and other annoyances incident to my condition, always occurred just as I was about to compose myself to sleep.

In the foregoing account, where I have given three, four, or five hours sleep, I would explain that I seldom had an unbroken repose for that time; that only on one occasion I had an hour's sleep in daylight; and that the night of the day on which I quitted the bed was the first I enjoyed for the entire night. In health, however, I am accustomed to little sleep—seldom more than five hours.

